# Autoantibodies against β_1_-adrenoceptor induce blood glucose enhancement and insulin insufficient via T lymphocytes

**DOI:** 10.1007/s12026-015-8757-7

**Published:** 2015-12-06

**Authors:** Yulin Gong, Haiyan Xiong, Yunhui Du, Ye wu, Suli Zhang, Xiao Li, Huirong Liu

**Affiliations:** Department of Physiology and Pathophysiology, School of Basic Medical Sciences, Capital Medical University, No. 10 Xi tou tiao, You An Men, Beijing, 100069 China; Beijing Key Laboratory of Metabolic Disorders Related Cardiovascular Disease, No. 10 Xi tou tiao, You An Men, Beijing, 100069 China

**Keywords:** Autoantibody against β_1_-adrenoceptors, Diabetes, Hyperglycemia, T lymphocytes, Insulin secretion

## Abstract

Diabetes mellitus is a chronic metabolic disorder with a high morbidity and mortality, but its pathogenesis is not fully understood. An increasing amount of evidence indicates that an immune mechanism plays an important role in the pathogenesis of diabetes. We demonstrated previously that the long-term presence of autoantibodies against the second extracellular loop of the β_1_-adrenoceptor (β_1_-AA) could change the ratio of peripheral CD4+T/CD8+T in rats, which was accompanied by lymphocytes infiltration in the rat heart, liver, and kidneys. To investigate whether β_1_-AA is involved in the pathogenesis of diabetes, *BALB/c* or nude mice were passively immunized with monoclonal antibodies against β_1_-AR (β_1_-AR mAb). Compared with vehicle control mice, β_1_-AA-positive *BALB/c* mice exhibited significantly increased blood glucose (*P* < 0.01) and increased fasting insulin (*P* < 0.05). However, the same changes did not occur in the nude mice. And altered islet morphology was found at week 28 in β_1_-AA immunization group compared with vehicle control. The basal insulin level of NIT-1 β-cells was decreased markedly (*P* < 0.01), and the lactate dehydrogenase level was increased (*P* < 0.01) after the administration of conditioned media from T lymphocytes that had been treated with β_1_-AA alone. However, these effects were reversed by treatment with metoprolol or peptides of the second extracellular loop of β_1_-adrenoceptor (β_1_-AR-EC_II_). These results suggest that β_1_-AA could induce hyperglycemia in both rats and mice, and also impair insulin secretion and change islet structure. T lymphocytes may play a key role in the pathogenesis of these changes in the islets.

**Electronic supplementary material:**

The online version of this article (doi:10.1007/s12026-015-8757-7) contains supplementary material, which is available to authorized users.

## Introduction

Diabetes mellitus is known as a chronic metabolic disease that is characterized by hyperglycemia and insulin resistance. According to the new data from the International Diabetes Federation (IDF), 387 million people are living with diabetes worldwide, and China has the largest number of people suffering from diabetes [1]. Therefore, diabetes causes a heavy burden in both China and worldwide. Diabetes is a complex syndrome that is the result of the combination of genetic factors and environmental stimuli [2, 3]; however, its pathogenesis is still not fully understood.

It is widely accepted that type 1 diabetes mellitus (T1DM) is an autoimmune disease that is characterized by the destruction of pancreatic β-cells, which causes an absolute deficiency of insulin secretion. An increasing number of notable discoveries in recent years have suggested that the immune system also plays an important role in the pathogenesis of type 2 diabetes mellitus (T2DM) [4–7]. A recent study reported that both the percentage of peripheral CD4+T cells and the ratio of CD4+T to CD8+T cells in T2DM patients were increased significantly, as well as the imbalance between regulatory T cells (Treg) and effector T cells (Teff). [8] Another study reported that proportion of circulating follicular helper T cells (CTfh) in peripheral CD4+T cells was increased significantly in T2DM patients than in controls [9]. A link between obesity, T2DM, and inflammation was established in the 1990s [10]. Subsequently, an increasing number of studies have demonstrated that as the resources of many different kinds of proinflammatory cytokines, T cells and immune system, both play a role in T2DM [11, 12]. Consistent with this, data that analyzed patients with prediabetes and T2DM showed that immunity-related biomarkers were altered dynamically during the progression of T2DM [13]. Similar immune changes also appear in animal models of diabetes; non-obese type 2 diabetes rats (Goto-Kakizaki rat) show higher levels of natural IgM and T cell ratios with elevated helper T(Th)2 cells compared with Wister rats. T cell ratios with elevated Th2 cells were observed in diabetic GK rats [14]. Taken together, these data suggest that T lymphocytes and the immune system contribute to the pathogenesis of both T1DM and T2DM.

Catecholamines can interfere with the function of T lymphocytes by activating β-adrenoceptors (β-ARs) [15, 16]. Furthermore, Yu et al. [17] demonstrated that β_1_-AR was expressed on the surface of T lymphocytes. Autoantibodies against the second extracellular loop of β_1_-adrenoceptors (β_1_-AAs; 100 % sequence identity between humans and rats) were first reported in 1987, and β_1_-AA acts similarly with agonist of β_1_-AR [18, 19]. We demonstrated previously that rats that had undergone long-term active immunization with the second extracellular loop of β_1_-AR (β_1_-AR-EC_II_) produced β_1_-AA and exhibited an elevated CD4+/CD8+T cells ratio, as well as lymphocyte infiltration into the heart, kidneys, and liver. [20] Taken together with observations that β_1_-AA enhanced the proliferation of T lymphocytes, this suggests that β_1_-AAs are an autoimmune product that participates in changes in multiple organs, thereby playing a key role in immunity in diabetes [21]. The aim of the current study was to investigate whether β_1_-AA is associated with diabetes, and whether T lymphocytes are implicated in this process.

## Materials and methods

### Animals

All animal experiments were performed in accordance with the Guide for the Care and Use of Laboratory Animals protocol, published by the Ministry of the People’s Republic of China (issued June 3, 2004) and were approved by the Institutional Committee on Animal Care of Capital Medical University. All *BALB/c* mice, *BALB/c* nude mice, and *Wistar* rats (180–220 g) used in the present study were obtained from the Animal Center of Capital Medical University. The mice were housed in pathogen-free conditions at 20–26 °C and were exposed to the conditions with 12/12-h light–dark periods daily. The mice and rats were fed rat chow and water ad libitum. The food was free from insulin and other oral glucose-lowering drugs.

### Peptide synthesis and active immunization

The peptide β_1_-AR-EC_II_ was synthesized as described previously using the sequence from the second extracellular loop of β_1_-AR (197–223 amino acid; H-W-W-R-A-E-S-D-E-A-R-R-C-Y-N-D-P-K-C-C-D-F-V-T-N-R-A), which shares 100 % homology between humans and mice [21]. Analytical high-performance liquid chromatography (HPLC) determined that the peptide preparations were 98 % pure. This work was performed by China Peptides Co. Ltd (Shanghai, China).

Eight-week-old healthy *Wistar* rats (weighing 180–200 g) that were sera-negative for β_1_-AA were divided randomly into two groups. Rats in the vehicle group (*n* = 15) were immunized with a mixture of normal saline and immunoadjuvant, whereas those in the β_1_-AA group (*n* = 15) were immunized with a mixture of artificial synthetic β_1_-AR-EC_II_ and immunoadjuvant. β_1_-AR-EC_II_ was dissolved in Na_2_CO_3_ solution (pH 11.0), mixed with the same volume of complete Freund’s adjuvant (Sigma-Aldrich, St. Louis, MO, USA), and then injected subcutaneously (SC) at a concentration of 0.4 μg/g posteriorly along the back at multiple sites (initial immunization). One week after the initial immunization, a booster SC injection of a mixture of an equal volume of peptide solution and incomplete Freund’s adjuvant (Sigma-Aldrich) was administered to one posterior site. A booster immunization was then given every 2 weeks subsequently. Specifically, rats in the vehicle group were injected SC with 1 mL saline mixed with 1 mL incomplete Freund’s adjuvant as described above. Blood samples were collected 1 day before each booster injection.

### Enzyme-linked immunosorbent assay (ELISA)

ELISAs were used to detect autoantibodies against β_1_-AR-EC_II_ in the sera of animals before and after immunization with β_1_-AR-EC_II_ or β_1_-AR mAb or vehicle, and the results were expressed as optical density (OD) units according to the methods published previously [22]. The antibody titer was also calculated according to the ratio of the OD values of positive and negative (P and N) controls as follows: ([Specimen OD − blank control OD]/[negative control OD − blank control OD]) [23].

Sera positive or negative for β_1_-AA were defined as samples with P/N ≥ 2.1 and P/N ≤ 1.5, respectively. The supernatants were then divided into small aliquots and stored for future use.

### Preparation of immunoglobulin G

Immunoglobulin G fractions (IgG) were prepared from the β_1_-AA-positive sera of active immunized *Wistar* rats or the ascites of *BALB/c* mice using MabTrap Kit (Amersham Bioscience, Uppsala, Sweden). The concentrations (mg/mL) and specificities of the purified IgGs were determined using a bicinchoninic acid (BCA) protein assay kit (Pierce, Rockford, USA) and ELISA as described above, respectively.

### Preparation of β_1_-AR monoclonal antibodies

Long and short peptides corresponding to amino acids 197–223 of the second extracellular loop of the human β_1_-AR were synthesized and then used to synthesize hybridoma that could produce monoclonal antibodies against β_1_-AR-EC_II_. This work was performed by a contractor (China Peptides). Anti-β_1_-AR-EC_II_ monoclonal antibodies (β_1_-AR mAb) were purified from hybridoma supernatants or the ascites of *BALB/c* mice using a MabTrap kit (Amersham Biosciences). The concentration of β_1_-AR mAb (mg/mL) and specificities were determined using a BCA Protein Assay kit (Pierce, Rockford, USA) and ELISA as described above, respectively.

### Passive immunization

*Passive immunization rat model*. The IgG was purified from the β_1_-AA-positive sera of active immunized rats. *Wistar* rats in the β_1_-AA group (*n* = 32) were injected with the purified IgG (0.7 μg/g) via the vena caudalis of rats every 2 weeks for 40 weeks, and four rats were died in the process of immunization. Rats in the vehicle group (*n* = 28) were injected with β_1_-AA-negative IgG via the same method. Blood samples were collected via the vena caudalis at weeks 0, 12, 24, and 36 for blood glucose measurements.

*Passive immunization of BALB/c mice and BALB/c nude mice*. *BALB/c* mice (*n* = 18) were injected intraperitoneally (IP) with β_1_-AR mAb (5 μg/g) every 2 weeks for 12 weeks; mice in the vehicle group (*n* = 18) were injected IP with the same volume of saline. *BALB/c* nude mice were administered as the same method of *BALB/c* mice, *n* = 18 each group.

### Intraperitoneal glucose tolerance test

Mice were fasted for 6 h before an IP glucose tolerance test (IGTT) was performed. An IP glucose solution (20 % glucose; 1 g glucose/kg bodyweight) was administered, and the blood glucose was monitored after 30, 60, and 120 min. The area under the curve (AUC) was determined by calculating the sum of rectangular area between each time point.

### Analysis of glucose, insulin, and glucagon

Mice and rats were fasted for at least 12 h but were allowed free access to water. Blood glucose levels were measured using an ACCU-CHEK active glucometer (Roche, Mannheim, Germany). Plasma insulin and glucagon were measured at 4-week intervals using an iodine [^125^I] insulin radioimmunoassay kit (F01PZB; Beijing North Institute of Biological Technology, China) or an iodine [^125^I] glucagon radioimmunoassay kit (F03PJA: Beijing North Institute of Biological Technology, China). Supernatant insulin of NIT-1 cells was measured using the same iodine [^125^I] insulin radioimmunoassay kit.

### Histopathological examination

Rats were killed after 24, 28, 32, and 36 weeks of passive immunization, and the pancreas was harvested, fixed in 10 % formaldehyde, and embedded in paraffin. The specimens were processed into 4-μm-thick paraffin sections, and the relative islet area was determined based on the method reported previously [24] after staining with hematoxylin and eosin (H&E).

### Cell lines and cell culture

NIT-1 cells, a pancreatic beta-cell line isolated from *NOD/Lt* mice, were purchased from Tongji Medical School, Huazhong University of Science and Technology, Wuhan, China [25]. They were cultured in RPMI1640 (Hyclone, Utah, USA) medium supplemented with 10 % FBS, 100 μg/mL streptomycin, and 100 U/mL penicillin [26]. T lymphocyte suspensions were prepared from the spleen of *BALB/c* mice as described previously [21]. T lymphocytes were incubated at 37 °C and 5 % CO_2_ for 24 h, and were then activated with 5 mg/L Concanavalin A (ConA) (Sigma-Aldrich). After activation, the T cells were seeded in 96-well plates and treated with saline (vehicle group), 0.1 μmol/L isoproterenol (positive control), 0.1 μmol/L β_1_-AA (β_1_-AA group), 0.1 μmol/L β_1_-AA + 3 μmol/L β_1_-AR-EC_II_, or 0.1 μmol/L β_1_-AA + 1 μmol/L metoprolol for 48 h. The supernatant was collected and added to NIT-1 cells for 6 or 24 or 48 h for measurement of insulin and LDH.

### Analysis of lactate dehydrogenase (LDH) levels

NIT-1 cells (1 × 10^4^ cells/well) were seeded in 96-well plate and cultured for 24 h. The media were then discarded and replaced with 100 μL of conditioned media collected as described above for another 6 or 12 or 24 or 48 h. LDH levels were then measured using commercially available kits (Beyotime, Shanghai, China).

### Statistical analysis

The results are presented as mean ± standard deviation (S.D), and Student’s *t* test was used to compare two independent sample means; one-way ANOVA was used to compare the means of more than two samples. All statistical analyses were performed using SPSS 13.0, and *P* ≤ 0.05 was considered to indicate significance (*). Nonsignificant differences are noted in the figures.

## Results

### β_1_-AA induced hyperglycemia in rats

**Fig. 1:**
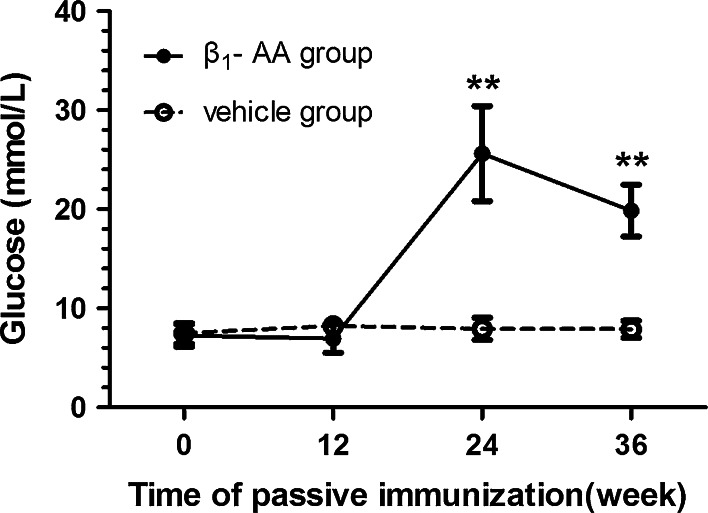
Blood glucose levels in the sera of rats after β_1_-AA passive immunization. Vehicle group (*n* = 32): rats were immunized with IgG from β_1_-AA-negative sera; β_1_-AA group (*n* = 28): rats passively immunized with β_1_-AA. Sera sample was collected and measured at weeks 0, 12, 24, 36 during the process of immunization. Data are presented as mean ± SD of three independent experiments. ^**^ *P* < 0.01 β_1_-AA group versus vehicle group at the same time point

The β_1_-AA-positive rat model was generated using passive immunization, which increased the blood glucose of the immunized rats gradually. After week 24 of immunization, the blood glucose of rats in the β_1_-AA group was significantly higher than that of rats in the vehicle group (24.6 ± 7.41 vs. 6.7 ± 0.30 mmol/L; *P* < 0.01) and reached a peak level (Fig. 1). The blood glucose level remained higher than the vehicle group at week 36 after immunization, suggesting that the long-term presence of β_1_-AA increased blood glucose.

### β_1_-AA increased blood glucose levels and impaired pancreatic function in vivo

**Fig. 2:**
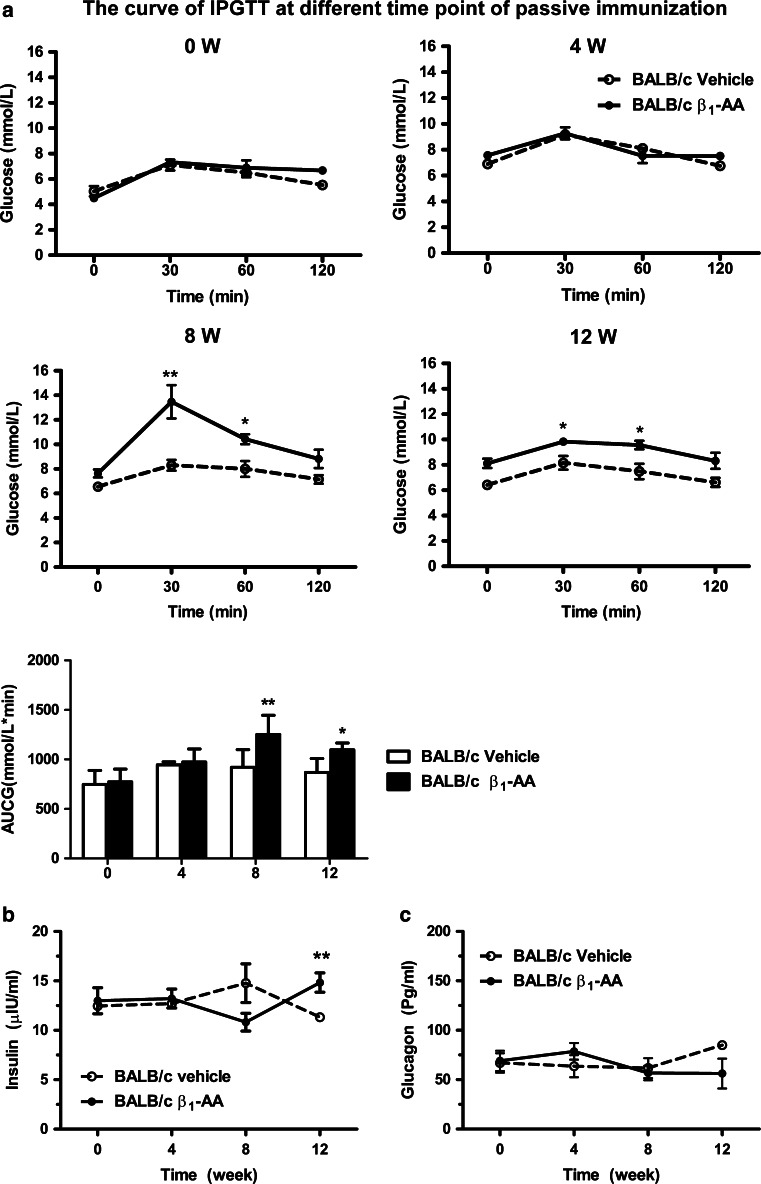
Changes in glucose, insulin, and glucagon in the process of *BALB/c* mice passive immunization. **a** IP GTT at different time points of passive immunization. *P < 0.05, **P < 0.01 β_1_-AA group versus vehicle group at the same time point. Fasting insulin (**b**) and glucagon (**c**) levels in the passive immunization. ^**^ *P* < 0.01 β_1_-AA *BALB/c* group versus vehicle *BALB/c* group at the same time point. Data are presented as mean ± SD of three independent experiments. *n* = 18/group

The results obtained from the β_1_-AA passive immunization rat model suggested that β_1_-AA increased blood glucose. To investigate the mechanism behind this change, a β_1_-AR mAb passive immunization *BALB/c* mouse model was generated (supplementary information). An IP GTT was performed on mice in both the β_1_-AR mAb and vehicle groups every 2 weeks from the beginning of passive immunization. There was no significant difference in the blood glucose levels of the two groups before the start of the experiment or after 4 weeks of immunization (Fig. 2a). However, GTTs performed after 8 and 12 weeks demonstrated that the blood glucose was increased in the β_1_-AR mAb group at 30 and 60 min compared with the vehicle group (week 8, 30 min: 13.47 ± 3.85 vs. 8.3 ± 1.39 mmol/L, respectively [*P* < 0.01]; 60 min: 10.43 ± 1.15 vs. 8 ± 2.03 mmol/L [*P* < 0.05]; week 12, 30 min: 9.83 ± 0.63 vs. 8.16 ± 1.32 mmol/L, respectively [*P* < 0.05]; 60 min, 9.57 ± 0.81 vs. 7.48 ± 1.50 mmol/L [*P* < 0.05]). The fasting insulin level was also measured in both groups. No significant differences were observed between during the first 8 weeks. However, fasting insulin was increased significantly in the β_1_-AR mAb group compared with the vehicle group on week 12 after immunization (14.83 ± 2.38 vs. 11.33 ± 0.75 μU/mL, respectively; *P* < 0.05; Fig. 2b). In contrast, there was no difference in glucagon between the β_1_-AR mAb and vehicle groups (Fig. 2c).

### β_1_-AA induced an irregular structure in pancreatic islets

**Fig. 3:**
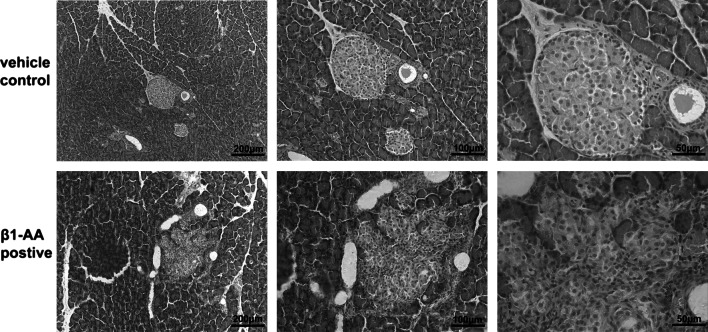
Morphology change in pancreatic islet. H&E staining of pancreatic islet on week 28 was measured by optical microscope. An irregular islet structure and decreased islet area were observed in the β_1_-AA group compared with vehicle. *Scale bar* of 100 times, 200 times, 400 times magnificatio*n* = 20, 10, 5 μm, respectively

The pancreases were harvested from passively immunized *Wistar* rats and analyzed using H&E staining. An irregular structure was observed in the β_1_-AR mAb group on week 28 (Fig. 3). Specifically, the islets exhibited an abnormal morphology, including unclear edges, a decreased area, and pancreatic acinar invasive into the islet. T lymphocyte infiltration was also observed, suggesting that β_1_-AA could disrupt the structure of the pancreatic islet and that T cells might be involved in this process.

### T lymphocytes mediated the β_1_-AA-induced hyperglycemia

**Fig. 4:**
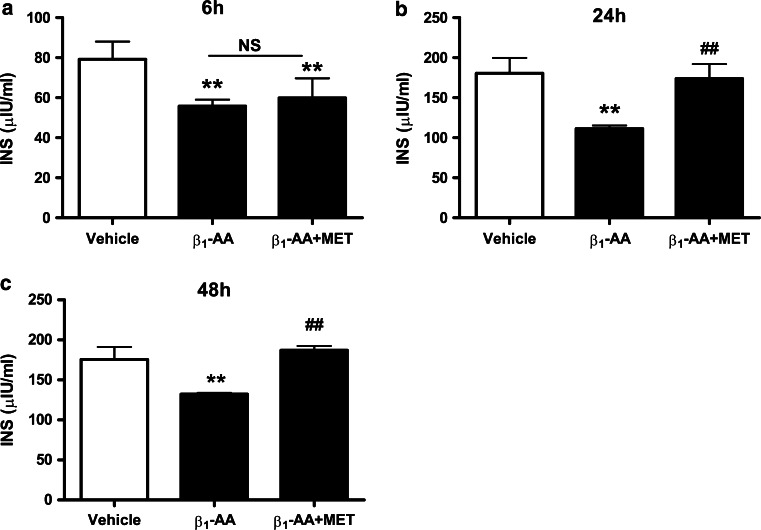
Insulin level of NIT-1 cells treated by supernatant of T cells stimulated by β_1_-AA or β_1_-AA+ metoprolol. Insulin level of NIT-1 cells after the treatment of T cells supernatant for 6 h (**a**), 24 h (**b**), 48 h (**c**).^**^ *P* < 0.01 versus vehicle group; ^##^ *P* < 0.01 versus β_1_-AA group. *MET* metoprolol. *n* = 6 in the 6 h group, and *n* = 4 in both the 24 and 48 h groups. Data are presented as mean ± SD of three independent experiments

**Fig. 5:**
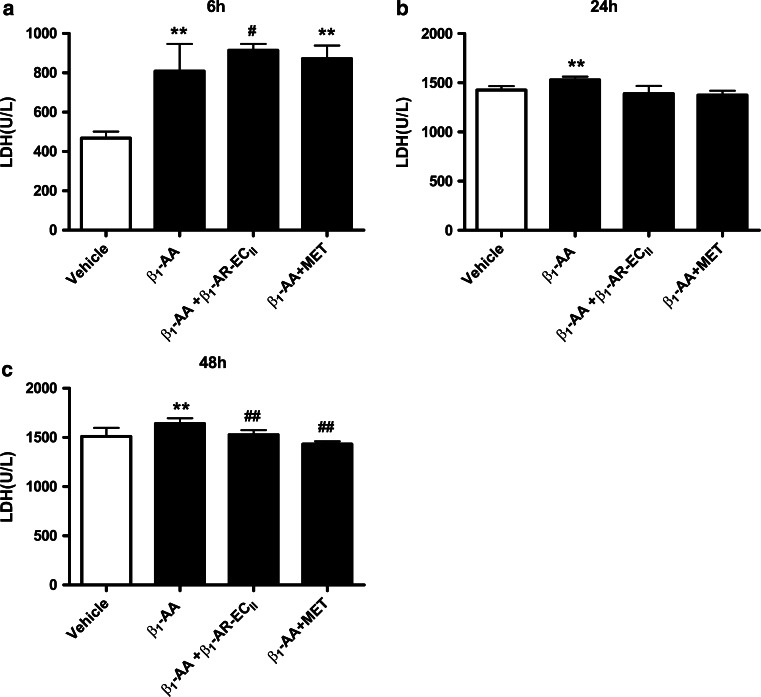
LDH level of NIT-1 cells treated by supernatant of T cells stimulated by β_1_-AA or β_1_-AA+ metoprolol or β_1_-AA+ β_1_-AR-EC_II_. LDH level of NIT-1 cells after the treatment of T cells supernatant for 6 h (**a**), 24 h (**b**), 48 h (**c**) ^**^ *P* < 0.01 versus vehicle group; ^#^ *P* < 0.05 versus β_1_-AA group; ^##^ *P* < 0.01 versus β_1_-AA group (*n* = 6/group). Data are presented as mean ± SD of three independent experiments

We next investigated the role of T cells in β_1_-AA-induced hyperglycemia. To test this, in vitro T lymphocytes were treated with β_1_-AA alone or β_1_-AA+ metoprolol for 48 h as described in method above. The supernatants were then harvested and used to treat NIT-1 cells for 6, 24, or 48 h. Insulin levels were decreased markedly in NIT-1 cells treated with β_1_-AA-stimulated T cell supernatant compared with those treated with vehicle-treated supernatant (6 h, 55.82 ± 3.23 vs. 79.23 ± 8.82 μU/mL, respectively [*P* < 0.01]; 24 h, 111.45 ± 3.91 vs. 180.58 ± 19.05 μU/mL [*P* < 0.01]; 48 h, 132.26 ± 1.64 vs. 175.52 ± 15.71 μU/mL [*P* < 0.01]; Fig. 4a–c). Metoprolol partially counteracted the effects of β_1_-AA on reducing insulin at 24 and 48 h, but not 6 h (*P* < 0.01). Furthermore, LDH assays demonstrated that incubation with T cell supernatant increased LDH levels of β_1_-AA group in 6, 24, and 48 h compared with vehicle group. (6 h, 808.37 ± 138.46 vs. 468.09 ± 32.82 U/L, respectively [*P* < 0.01]; 24 h, 1528.67 ± 33.82 vs. 1426.67 ± 39.41 U/L, [*P* < 0.01]; 48 h, 1641.04 ± 54.91 vs. 1511.38 ± 85.56 U/L, [*P* < 0.05]; Fig. 5a–c) And the LDH level of β_1_-AA group in 24 and 48 h was apparently higher than 6 h. However, the presence of the β_1_-AR blocker metoprolol or β_1_-AR-EC_II_ suppressed the increasing LDH levels compared with β_1_-AA alone at 6 and 48 h, but not 24 h (6 h, 808.37 ± 138.46 vs. β_1_-AA + β_1_-AR-EC_II_ group 914.55 ± 32.25 U/L, [*P* < 0.05]; 48 h, 1641.04 ± 54.91 vs. β_1_-AA + β_1_-AR-EC_II_ group 1527.04 ± 47.18 U/L, [*P* < 0.01], 1641.04 ± 54.91 vs. β_1_-AA + metoprolol group 1432.99 ± 27.91 U/L, [*P* < 0.01]; Fig. 5a, c).

### β_1_-AA did not cause glucose imbalance and pancreatic dysfunction in nude mice

**Fig. 6:**
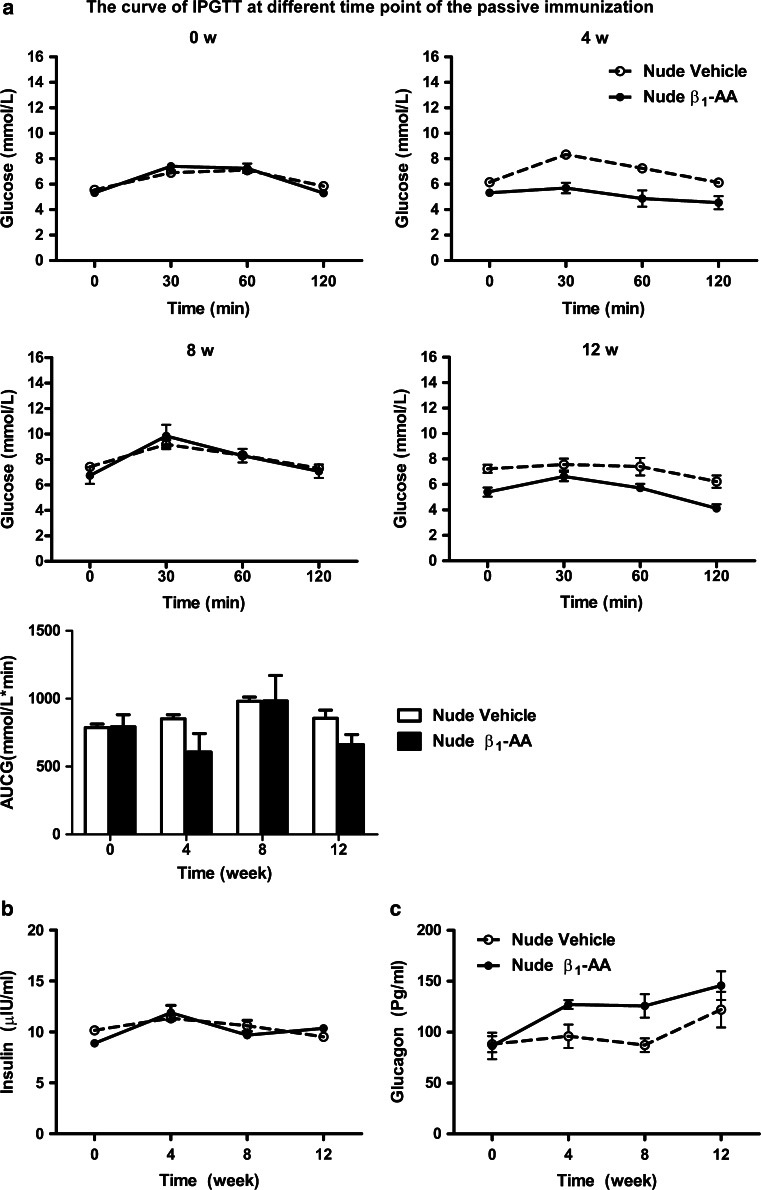
Changes in glucose, insulin, and glucagon levels during the passive immunization of nude *BALB/c* mice. **a** IP GTT curve after different weeks of passive immunization in nude mice. There were no significant difference between the β_1_-AA group and vehicle groups at any time points (*n* = 18/group). **b** No significant differences in insulin levels in *BALB/c* nude mice between the vehicle and immunized groups (*n* = 18/group). **c** No significant differences in glucagon levels in *BALB/c* nude mice between the vehicle and immunized groups (*n* = 18/group). Data are presented as mean ± SD of three independent experiments

The in vitro results demonstrated that T cells might contribute to the β_1_-AA-induced increased glucose levels. Nude mice lack the thymus and so are often used in immune-related research. Therefore, we generated a β_1_-AR mAb passive immunization model in *BALB/c* nude mice and measured IP GTT, and fasting insulin and glucagon levels. There were no significant differences in blood glucose, fasting insulin, or glucagon levels between the β_1_-AR mAb and vehicle groups at any time after immunization (Fig. 6a–c). These results demonstrated that the increased blood glucose levels and pancreatic islet dysfunction observed in β_1_-AR mAb passively immunized *BALB/c* mice did not occur in nude mice. This suggests that T lymphocytes might play a role in the glucose imbalance and pancreatic islet injury induced by β_1_-AA.

## Discussion

Diabetes is a serious chronic metabolic disease. In addition to the disease itself, the complications caused by diabetes further increase the mortality and morbidity rates. Over time, diabetes can damage the heart, blood vessels, eyes, kidneys, and nerves. However, the mechanisms underlying diabetes have not yet been elucidated fully. The current study introduced a novel mechanism behind diabetes that is caused by autoantibodies against β_1_-AA. Most studies into β_1_-AA have focused on cardiovascular diseases, including heart failure, idiopathic cardiomyopathy, and arrhythmia [18, 27–29]. In addition, recent studies reported that β_1_-AA could induce injury in the kidneys and heart [20, 30]. These results suggest that the impairments caused by β_1_-AA might not being limited to the cardiovascular system, but are instead more likely affect multiple systems. The current findings of increased blood glucose levels and pancreatic injury are consistent with this viewpoint.

The current study demonstrated that passive immunization with β_1_-AA increased blood glucose in both rats and mice. In the passive immunization rat model, a time-dependent increase in blood glucose levels was observed during the immunization period, and levels peaked at week 24. But in the β_1_-AR mAb passive immunization *BALB/c* mice model, increased blood glucose was observed on week 8, which is earlier than the week-24 increase observed in the β_1_-AA-positive IgG immunization rats model. It is possible that β_1_-AA might affect glucose homeostasis before diabetes diagnosis. Importantly, impaired glucose tolerance (IGT) is the stage before diabetes, and individuals with IGT or IFG are at a high risk of progressing to T2DM [31]. Taken together, these observations suggest that IGT might occur before β_1_-AA-induced increased blood glucose levels. Therefore, we used IP GTTs to examine glucose tolerance and glucose levels. Furthermore, fasting insulin levels were similar between groups on week 4, but a trend toward a reduced insulin level was observed in the β_1_-AA group at week 8. On week 12, insulin levels were increased significantly in the β_1_-AA group compared with the vehicle group. This is consistent with the variations in insulin levels observed in diabetic patients [32], which suggests that β_1_-AA could induce insulin compensation in mice.

IgG purified from the sera of β_1_-AA-positive rats was used to generate a passive immunization rat model. The IgGs used included specific antibodies against the β_1_-AR-EC_II_ as well as other IgGs; therefore, other kinds of antibodies might have interfered with the effects of β_1_-AA. Therefore, we synthesized β_1_-AR mAb, a monoclonal antibody from mice that eliminates nonspecific IgGs and other effects.

In a β_1_-AR-EC_II_ long-term active immunization model, Zuo reported that the CD4+/CD8+T cells’ ratio was elevated compared with vehicle control [20]. Another previous study revealed that β_1_-AA from dilated cardiomyopathy (DCM) patients enhanced the proliferation of T lymphocytes [21]. This suggests that changes in the T lymphocyte population might accompany the presence of β_1_-AA. The current observations of pancreatic morphology also support this hypothesis, since lymphocyte infiltration in the pancreas was also observed during β_1_-AA immunization. In addition to the important role of T lymphocytes in the pathogenesis of diabetes, we also assessed whether T lymphocytes play a role in β_1_-AA-induced high blood glucose levels using a β_1_-AA passive immunization model in nude mice. There were no differences in blood glucose, insulin, or glucagon levels between the β_1_-AA and vehicle groups, suggesting that T lymphocytes play an important role in β_1_-AA-induced pancreatic dysfunction. However, T lymphocytes are not the only reason for β_1_-AA-induced elevated blood glucose levels. According to our observations in NIT-1 cells in vitro, increased LDH and decreased insulin levels could be partially counteracted by metoprolol or β_1_-AR-EC_II_, suggesting that the effects of β_1_-AA on β-cell dysfunction were exerted either via β_1_-ARs on T cells or directly actions on β-cells. The decrease in insulin level in 24 and 48 h was severe than 6 h, and the decrease could be counteracted by β-blocker metoprolol, the probable reason of this phenomenon is the long-term effect of β_1_-AA. As far as we know, the injury caused by β_1_-AA in various kinds of cardiac disease is a long time consequence [20, 33–35]. With the stimulation prolonged, the injury becomes more severe; thus, the protective effect of metoprolol seems more obviously. The increase in LDH in 24-h group did not counteract by metoprolol or β_1_-AR-EC_II_. As far as we know, there was very rare research about β_1_-AA and metabolic disease including diabetes. The only two articles in Chinese were focusing on the presence of β_1_-AA in type 2 diabetes with hypertension [36, 37]. Taken together, the current study, for the first time, revealed that long-term exposure to β_1_-AA increased blood glucose levels and impaired insulin secretion. Furthermore, T lymphocytes were implicated in this process, but T cells may not be the only element participant in it.

## Electronic supplementary material

Supplementary material 1: The β_1_-AA passive immunization models in *BALB/c* mice and *BALB/c* nude mice were established successfully. The β_1_-AA serum titers were increased significantly increased compared with the vehicle group. ^*^ *P*<0.05 β_1_-AA group vs. vehicle group at the same time point. Data are presented as means + SD of 3 independent experiments, n=18 each group. (TIFF 225 kb)

## Abbreviations

DM: Diabetes mellitus
β_1_-AR: Β_1_-adrenoceptor
β_1_-AR-EC_II_: The second extracellular loop of β_1_-AR
β_1_-AR mAb: Monoclonal antibody against β_1_-AR-EC_II_
β_1_-AA: Autoantibodies against the second extracellular loop of β_1_-AR
T1DM: Type 1 diabetes mellitus
T2DM: Type 2 diabetes mellitus
DCM: Dilated cardiomyopathy
IPGTT: Intraperitoneal glucose tolerance test
AUC: Area under the curve
LDH: Lactate dehydrogenase
IDF: International Diabetes Federation
Treg: Regulator T cells
Teff: Effector T cells
CTfh: Circulating follicular helper T cells
Th: Helper T cells
GK rat: Goto-Kakizaki rat
HPLC: High-performance liquid chromatography
SC: Subcutaneously
ELISA: Enzyme-linked immunosorbent assay
OD: Optical density
IgG: Immunoglobulin G fractions
ConA: Concanavalin A

## References

[1] IDF diabetes ATLAS six edition. International Diabetes Federation, 2013.

[2] Hewagama A, Richardson B (2009). The genetics and epigenetics of autoimmune diseases. J Autoimmun.

[3] Andreassi MG (2009). Metabolic syndrome, diabetes and atherosclerosis: influence of gene-environment interaction. Mutat Res.

[4] Ehses JA (2009). Pancreatic islet inflammation in type 2 diabetes: from alpha and beta cell compensation to dysfunction. Arch Physiol Biochem.

[5] Donath MY (2009). Islet inflammation impairs the pancreatic beta-cell in type 2 diabetes. Physiology (Bethesda).

[6] Butcher MJ (2014). Association of proinflammatory cytokines and islet resident leucocytes with islet dysfunction in type 2 diabetes. Diabetologia.

[7] Brooks-Worrell BM, Boyko EJ, Palmer JP (2014). Impact of islet autoimmunity on the progressive beta-cell functional decline in type 2 diabetes. Diabetes Care.

[8] Cai B (2013). Micro-inflammation characterized by disturbed Treg/Teff balance with increasing sIL-2R in patients with type 2 diabetes. Exp Clin Endocrinol Diabetes.

[9] Wang Q (2015). Dysregulation of circulating CD4+CXCR5+T cells in type 2 diabetes mellitus. APMIS.

[10] Hotamisligil GS, Shargill NS, Spiegelman BM (1993). Adipose expression of tumor necrosis factor-alpha: direct role in obesity-linked insulin resistance. Science.

[11] Dai X (2015). Monocytes play different roles in stimulating T cells in obese diabetic individuals. Int J Immunopathol Pharmacol.

[12] Seijkens T (2014). Immune cell crosstalk in obesity: a key role for costimulation?. Diabetes.

[13] Grossmann V (2015). Profile of the immune and inflammatory response in individuals with prediabetes and type 2 diabetes. Diabetes Care.

[14] Takeda Y, Shimomura T, Wakabayashi I (2014). Immunological disorders of diabetes mellitus in experimental rat models. Nihon Eiseigaku Zasshi.

[15] Jiang JL (2009). Effect of the endogenous catecholamines synthesized by lymphocytes on T cell proliferation. Zhongguo Ying Yong Sheng Li Xue Za Zhi.

[16] Luecken LJ, Lysle DT (1992). Evidence for the involvement of beta-adrenergic receptors in conditioned immunomodulation. J Neuroimmunol.

[17] Yu XY (2007). Evidence for coexistence of three beta-adrenoceptor subtypes in human peripheral lymphocytes. Clin Pharmacol Ther.

[18] Wallukat G, Wollenberger A (1987). Effects of the serum gamma globulin fraction of patients with allergic asthma and dilated cardiomyopathy on chronotropic beta adrenoceptor function in cultured neonatal rat heart myocytes. Biomed Biochim Acta.

[19] Wallukat G (1995). Anti-beta 1-adrenoceptor autoantibodies with chronotropic activity from the serum of patients with dilated cardiomyopathy: mapping of epitopes in the first and second extracellular loops. J Mol Cell Cardiol.

[20] Zuo L (2011). Long-term active immunization with a synthetic peptide corresponding to the second extracellular loop of beta1-adrenoceptor induces both morphological and functional cardiomyopathic changes in rats. Int J Cardiol.

[21] Du Y (2012). beta1-Adrenoceptor autoantibodies from DCM patients enhance the proliferation of T lymphocytes through the beta1-AR/cAMP/PKA and p38 MAPK pathways. PLoS ONE.

[22] Liu HR (1999). Screening of serum autoantibodies to cardiac beta1-adrenoceptors and M2-muscarinic acetylcholine receptors in 408 healthy subjects of varying ages. Autoimmunity.

[23] Liu HR (2002). Relationship of myocardial remodeling to the genesis of serum autoantibodies to cardiac beta(1)-adrenoceptors and muscarinic type 2 acetylcholine receptors in rats. J Am Coll Cardiol.

[24] Butler AE (2003). Beta-cell deficit and increased beta-cell apoptosis in humans with type 2 diabetes. Diabetes.

[25] Hamaguchi K, Gaskins HR, Leiter EH (1991). NIT-1, a pancreatic beta-cell line established from a transgenic NOD/Lt mouse. Diabetes.

[26] Zhao P, Yang X (2013). Vanadium compounds modulate PPARgamma activity primarily by increasing PPARgamma protein levels in mouse insulinoma NIT-1 cells. Metallomics.

[27] Kaya Z, Leib C, Katus HA (2012). Autoantibodies in heart failure and cardiac dysfunction. Circ Res.

[28] Iwata M (2001). Autoantibodies against the second extracellular loop of beta1-adrenergic receptors predict ventricular tachycardia and sudden death in patients with idiopathic dilated cardiomyopathy. J Am Coll Cardiol.

[29] Zhao Y (2015). beta1-Adrenoceptor autoantibodies affect action potential duration and delayed rectifier potassium currents in guinea pigs. Cardiovasc Toxicol.

[30] Zuo L (2014). Presence of autoantibodies against beta1-adrenoceptor aggravates the kidney injury in rats. Sheng Li Xue Bao.

[31] Geneva WHO. Definition, diagnosis and classification of diabetes mellitus and its complications. Part 1: Diagnosis and classification of diabetes mellitus. WHO; 1999.

[32] Stancakova A (2009). Changes in insulin sensitivity and insulin release in relation to glycemia and glucose tolerance in 6,414 Finnish men. Diabetes.

[33] Dandel M (2012). Role of beta(1)-adrenoceptor autoantibodies in the pathogenesis of dilated cardiomyopathy. Immunobiology.

[34] Brisinda D (2012). Anti-beta-adrenoceptors autoimmunity causing ‘idiopathic’ arrhythmias and cardiomyopathy. Circ J.

[35] Iwata M (2001). Autoimmunity against the second extracellular loop of beta(1)-adrenergic receptors induces beta-adrenergic receptor desensitization and myocardial hypertrophy in vivo. Circ Res.

[36] Zhao LS (2010). Positive rate of autoantibodies against adrenergic receptors beta1 and angiotensin II type 1 receptors in the type 2 diabetes mellitus with or without hypertension. Zhonghua Xin Xue Guan Bing Za Zhi.

[37] Zhao LS (2006). Autoantibodies against beta1 and M2 receptor in diabetic patients with refractory hypertension. Zhonghua Xin Xue Guan Bing Za Zhi.

