# Supplementary figures and images for: Autoantibodies against β1-adrenoceptor induce blood glucose enhancement and insulin insufficient via T lymphocytes
Source: Immunol Res. 2015 Dec 6;64:584–93. doi: 10.1007/s12026-015-8757-7 (PMC4788697; doi:10.1007/s12026-015-8757-7)

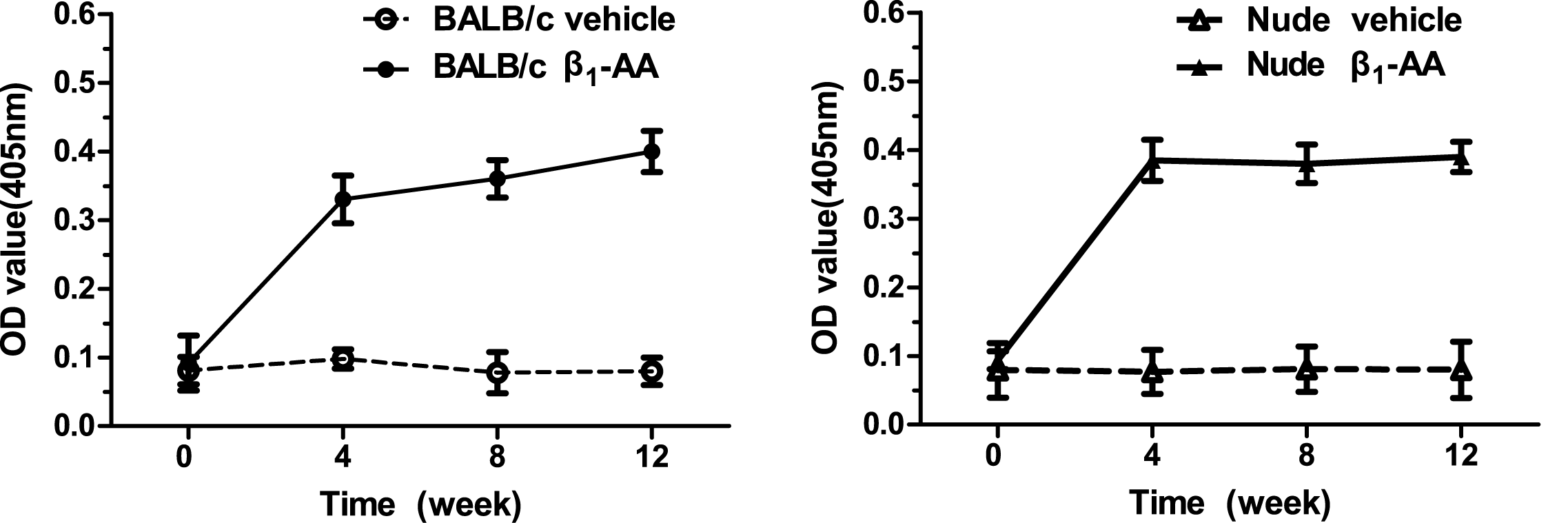

Supplement: Supplementary file 1 — Supplementary material 1: The β1-AA passive immunization models in BALB/c mice and BALB/c nude mice were established successfully. The β1-AA serum titers were increased significantly increased compared with the vehicle group. * P<0.05 β1-AA group vs. vehicle group at the same time point. Data are presented as means + SD of 3 independent experiments, n=18 each group. (TIFF 225 kb) [file 12026_2015_8757_MOESM1_ESM.tif]
